# Use of a fixed combination of acetylsalicylic acid, acetaminophen and caffeine compared with acetaminophen alone in episodic tension-type headache: meta-analysis of four randomized, double-blind, placebo-controlled, crossover studies

**DOI:** 10.1186/1129-2377-15-76

**Published:** 2014-11-19

**Authors:** Hans-Christoph Diener, Morris Gold, Martina Hagen

**Affiliations:** 1 Department of Neurology, University Hospital Essen, University Duisburg-Essen, Hufelandstr. 55, 45122 Essen, Germany; 2 Novartis Consumer Health Inc., Parsippany, NJ, USA; 3 Novartis Consumer Health S.A., Nyon, Switzerland

**Keywords:** Acetaminophen, Acetylsalicylic acid, Caffeine, Episodic tension-type headache, Severe pain, Triple combination

## Abstract

**Background:**

Most patients with episodic tension-type headache treat headache episodes with over-the-counter medication. Combination analgesics containing caffeine may be more effective and as well tolerated as monotherapy. The aim of this study was to evaluate the efficacy of the combination of acetylsalicylic acid, acetaminophen (paracetamol) and caffeine in episodic tension-type headache using recently recommended endpoints.

**Methods:**

Four randomized, controlled trials of identical design in 1,900 patients with episodic tension-type headache comparing acetylsalicylic acid, acetaminophen and caffeine vs. acetaminophen or placebo were pooled. Analysis populations were ‘all headache episodes’ and those with ‘severe pain at baseline’. Post-hoc defined primary endpoint: headache episodes pain-free at 2 h. Secondary endpoints: headache episodes pain-free at 1 h, headache response at 2 h (mild or no pain), degree of interference with daily activities.

**Results:**

6,861 headache episodes were treated, including 2,215 severe headache episodes. The proportion of headache episodes pain-free at 2 h was significantly higher with the triple combination (28.5%) vs. acetaminophen (21.0%) and placebo (18.0%) (p < 0.0001), and similarly for those severe at baseline (20.2% vs. 12.1% and 10.8%; p ≤ 0.0003). A similar pattern of superiority was observed for secondary endpoints. The triple combination was generally well tolerated.

**Conclusions:**

The combination of acetylsalicylic acid, acetaminophen and caffeine is effective and well tolerated in episodic tension-type headache, and significantly superior to acetaminophen with regard to being pain-free at 2 h, headache response at 2 h and ability to return to daily activities, even in those with pain rated severe at baseline.

## Background

Tension-type headache (TTH) is by far the most prevalent primary headache [1,2]. In general, most patients with episodic TTH never consult a physician [3] and treat the headache episodes with over-the-counter (OTC) medication [4]. The most frequently used drugs are acetylsalicylic acid (ASA), acetaminophen (APAP; paracetamol) and non-steroidal anti-inflammatory drugs (NSAIDs) such as ibuprofen. In fact, such drugs are the only options available, as specific migraine medications such as triptans are not effective in TTH [5], while opioids increase the risk of medication-overuse headache [6]; neither is recommended for use in TTH [7]. However, some patients or headache episodes might not respond to monotherapy, and patients may overuse an analgesic in order to relieve their pain. Therefore, combination therapies with ASA, APAP and caffeine were developed to provide superior efficacy using lower doses of analgesics compared with therapeutic doses of monotherapy [8,9].

In this paper, we describe four multicenter, randomized, double-blind, crossover studies comparing the effectiveness of a single dose of a combination analgesic containing ASA, APAP and caffeine vs. APAP alone or placebo in patients with moderate or severe episodic TTH. These studies were pooled for a meta-analysis of the efficacy of the triple combination. The original studies were conducted in the 1980s, and the results were published by Migliardi et al. [10]. The purpose of the Migliardi paper was to evaluate the effect of caffeine in TTH and therefore included the results of all studies in TTH that used an analgesic–caffeine combination. However, we wanted to concentrate only on the efficacy of the triple combination (ASA, APAP and caffeine) and hence only included four of the studies assessed in the Migliardi paper [10]. Furthermore, the efficacy analysis was updated to reflect endpoints that have recently been recommended to better differentiate among treatments in clinical trials of TTH, i.e. pain-free after 2 h (Y/N) and headache response after 2 h (Y/N) [11,12]. These endpoints are particularly appropriate to guide therapeutic choices [11]. In addition, it was decided to see how effective the triple combination was in the subset of patients with severe pain at baseline.

## Methods

### Original studies

#### Subjects

Adults aged 18 to 65 years of either sex diagnosed with episodic TTH were randomized into the four studies. The diagnostic criteria for muscle contraction or tension headache were those that were commonly recognized at the time the study was conducted, i.e.:

a. Ache or sensation of tightness, pressure, or constriction, widely varied in intensity, frequency, and duration, sometimes long-lasting [13].

b. Ache or pain associated with contraction of neck, face, and scalp muscles in the absence of permanent structural changes; usually as part of the individual’s reaction to life stress.

c. Headache described as being a steady ache or as a “tightness”, “pressure”, “drawing”, or soreness of the head.

d. Headache, commonly bilateral (sometimes unilateral), at center near occiput and posterior cervical region that may radiate to the temples, jaws, portions of the face or vertex cranii or constitute a “bandlike” constriction about the head.

e. Tenderness of the trapezius or posterior cervical muscles on palpation may be present. At times localized tender, taut muscle fibers may be noted. Pressure on these areas may cause pain to spread to adjacent portions of the head or face.

These criteria fulfil at least two of the current diagnostic requirements for episodic TTH (i.e. bilateral location, pressing/tightening (non-pulsating) quality), but no indication was given regarding the frequency or duration of headaches [14]. In addition, the presence of other symptoms (e.g. nausea, photophobia, phonophobia) was not recorded. All patients were in good general health, as determined by medical history and review of current medications. Eligible patients averaged at least four but no more than ten tension-type headache episodes per month during the last year, which usually responded to OTC analgesics. Patients with a history of chronic, recurrent or continuous headache episodes, vascular headache of migraine type, post-traumatic headache or other types of headache were excluded. Institutional Review Board approval for each center was obtained and all subjects provided written, informed consent. All studies were conducted according to the ethical principles of the Declaration of Helsinki.

#### Interventions

The three treatments administered in the studies were a triple combination analgesic (unbranded Extra-Strength Excedrin®, Novartis Consumer Health; two tablets, each containing ASA 250 mg, APAP 250 mg and caffeine 65 mg) (AAC), APAP alone (unbranded Tylenol®, McNeil Consumer Healthcare; two 500 mg caplets) and placebo. Blinding was achieved through double-dummy dosing. Each TTH attack was treated with a single dose of study medication. Any rescue medication (subjects’ usual medication), as well as any medication required to treat adverse events (AEs) during the 4 h evaluation period was recorded on a self-rating record. No other medication of any kind was taken concurrently with the study medication.

#### Study design

**Figure 1:**
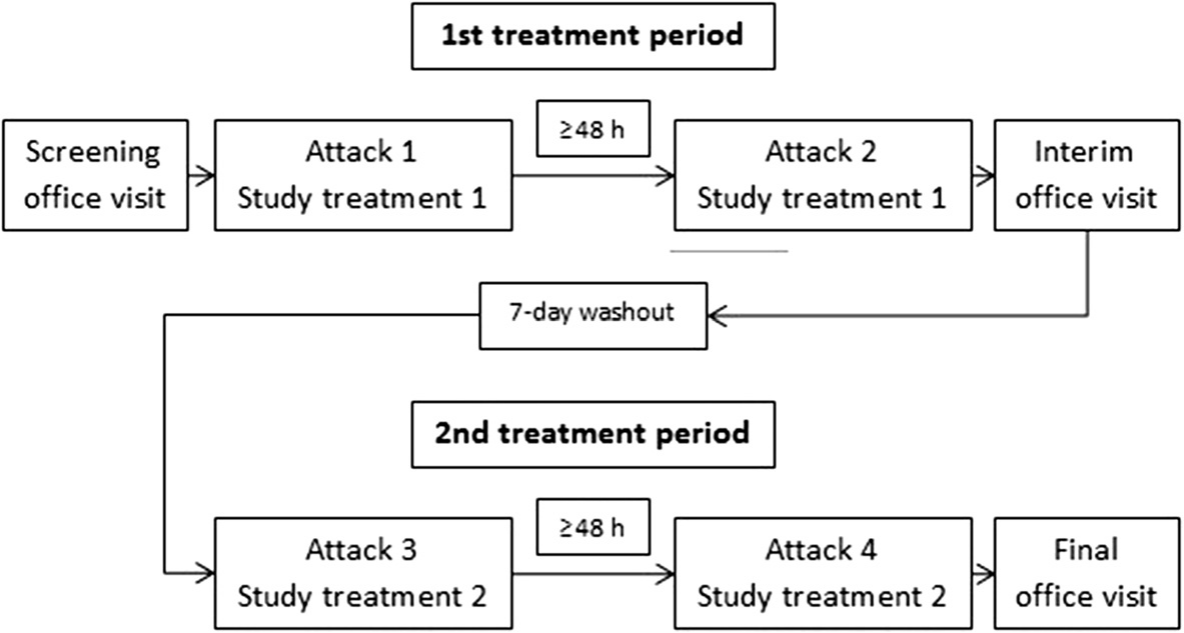
Design of the four randomized, controlled studies included in the meta-analysis.

The same protocol was used for each of the four studies. In the common incomplete block crossover study design, each subject was randomized to one of six possible sequences, in which two of the three treatments were taken over the two periods (Figure 1). The randomization was weighted so that overall, twice as many patients dosed with each of the two active treatments as with placebo. The two periods were separated by a washout of at least 7 days, during which subjects took their own usual medication for any headache episodes or other pain.

Within each period, subjects treated two TTH attacks with single doses of the study medication designated for that period by the randomized treatment sequence. The two treated headache episodes were separated by at least 48 h. Subjects were instructed to treat only TTH headache episodes with at least moderate pain severity. At baseline, subjects recorded the amount of any caffeine-containing foods and beverages consumed in the preceding 4 h, administered a single 4-tablet dose of the study medication and then abstained from caffeine-containing foods and beverages for the following 4 h. Further details of the design can be found in the original publication of these studies by Migliardi et al. [10].

#### Efficacy parameters

After dosing, subjects evaluated pain intensity hourly for 4 h on a 4-point scale (0 = none; 1 = mild; 2 = moderate; 3 = severe). The degree of interference with daily activities was also measured on a 4-point scale (0 = no more difficult than usual, 1 = require some additional effort, 2 = more difficult than usual, 3 = impossible).

#### Data adjustment

Concomitant medications that could interfere with the scoring of the efficacy variables were taken into consideration before analysis: all pain and discomfort scores recorded more than 15 minutes after the subject remedicated were replaced by either the score measured at baseline or by the last score measured before remedication, whichever was less favorable.

#### Safety analysis

Adverse events were coded according to an AE coding dictionary that was current at the time the studies were conducted. Each AE was attributed to the study treatment taken mostly recently before the AE occurred.

### Meta-analysis

#### Data presentation and analysis

The primary endpoint of the present meta-analysis was the percentage of treated headache episodes in which subjects were pain-free 2 h after dosing. This outcome is recommended by the International Headache Society (IHS) to be primary in trials that utilize the TTH clinical model [12]. Secondary endpoints were the percentage of treated headache episodes pain-free 1 h after dosing and headache response 2 h after dosing (defined as a reduction in headache intensity from moderate or severe pain to mild or no pain). We also compared the treatments at each hour on the degree of interference with daily activities, specifically the percent of patients who responded ‘no more difficult than usual’. Results are presented for ‘all headache episodes’, as well as for the subset with ‘severe pain at baseline’. The safety data was taken directly from Migliardi et al. [10].

#### Statistical methods

The statistical methods by which the individual studies were analyzed are described in Migliardi et al. [10]. The efficacy evaluable (EE) population for each study was defined as all subjects who treated at least one headache in both treatment periods.

For each binary outcome (pain-free [Y/N], having headache response [Y/N] and whether the degree of interference with daily activities was “no more difficult than usual” [Y/N]), simple percent success rates were computed at each hour for each treatment over all headache episodes experienced by all subjects pooled over the four studies who dosed with that treatment.

To address the within-subject correlation imposed by the crossover design, efficacy with respect to binary outcomes was tested in a conditional logistic regression model, stratified by subject, having main effects of period and treatment, with baseline pain severity as a covariate and with the interaction of period and the baseline covariate. The two-way interactions of treatment with both period and the baseline covariate were tested, but were not found to be statistically significant and were dropped from the model. Pairwise tests for differences in efficacy between treatments were based on the least squares estimates of the pairwise odds ratios. All tests were two-sided and p < 0.05 was the cutoff for statistical significance. All efficacy analyses were done in SAS for Windows Version 9.3 (SAS Institute Inc., Cary, NC).

## Results

### Characteristics of included studies

**Figure 2:**
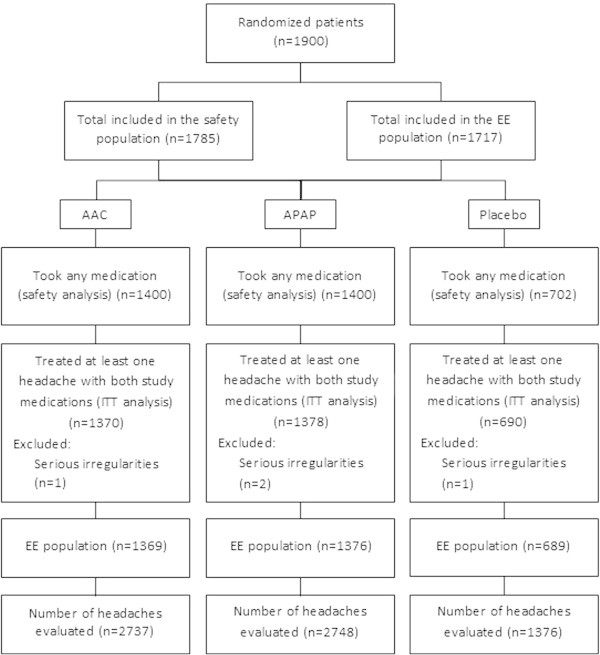
**Summary flowchart of the patients included in the meta-analysis.** Safety analysis data taken from Migliardi et al. [10]; EE = efficacy evaluable; ITT = intention-to-treat.

In total, 1,900 patients were randomized by 36 investigators (Figure 2). Of these, 1,785 patients used study medication at least once and 1,719 took at least one dose of study medication in both treatment periods. However, two subjects were excluded because of serious irregularities: one received the same treatment in both periods and the other provided dates of dosing in the second treatment period that were earlier than those in the first treatment period. Thus, 1,717 patients were included in the EE analysis. The EE population is therefore trivially different from the maximal population for efficacy analysis. The EE analysis comprised: 1,369 AAC patients who treated 2,737 headache episodes; 1,376 APAP patients who treated 2,748 headache episodes; 689 placebo patients who treated 1,376 headache episodes. In 32% of treated episodes, severe pain was reported at baseline (Table 1). The demographics of all patients included are described in Table 1. Apart from headache intensity, the patients with severe pain at baseline had similar baseline characteristics to those with moderate pain.

**Table 1:** Pooled characteristics of all patients included in the efficacy evaluable population (i.e. all subjects who treated at least one headache in both treatment periods) in the four randomized, controlled studies included in the meta-analysis

**Characteristic**	**AAC**	**APAP**	**Placebo**	**Total**
Number of patients	1369	1376	689	1717
Number of headache episodes treated	2737	2748	1376	6861
Mean age (SD), y	34.1 (9.7)	34.1 (9.7)	34.5 (10.0)	34.2 (9.8)
Sex (F), n (%)	1129 (82.5)	1140 (82.9)	563 (81.7)	1416 (82.5)
Race, n (%)				
Caucasian	1238 (90.4)	1245 (90.5)	625 (90.7)	1554 (90.5)
Other	131 (9.6)	131 (9.5)	64 (9.3)	163 (9.5)
Mean no. headache episodes/month (SD)	6.4 (2.1)	6.4 (2.1)	6.4 (2.1)	6.4 (2.1)
Mean age at onset (SD), y	22.1 (8.7)^a^	22.2 (8.6)^b^	21.9 (8.6)^c^	22.1 (8.6)^d^
Onset, n (%)				
Gradual	983 (71.9)	989 (71.9)	478 (69.4)	1225 (71.4)
Rapid	385 (28.1)	386 (28.1)	211 (30.6)	491 (28.6)
Location, n (%)				
Suboccipital	288 (21.0)	285 (20.7)	133 (19.3)	353 (20.6)
Occipital	411 (30.0)	388 (28.2)	201 (29.2)	500 (29.1)
Parietal	95 (6.9)	98 (7.1)	49 (7.1)	121 (7.1)
Temporal	640 (46.8)	659 (47.9)	309 (44.9)	804 (46.8)
Frontal	750 (54.8)	759 (55.2)	385 (55.9)	947 (55.2)
Whole head	205 (15.0)	205 (14.9)	106 (15.4)	258 (15.0)
Character, n (%)				
Non-throbbing	404 (29.5)	397 (28.9)	207 (30.0)	504 (29.4)
Throbbing	341 (24.9)	343 (24.9)	182 (26.4)	433 (25.2)
Combination	624 (45.6)	636 (46.2)	300 (43.5)	780 (45.4)
Usual severity (history), n (%)				
Mild	12 (0.9)	8 (0.6)	8 (1.2)	14 (0.8)
Moderate	789 (57.6)	784 (57.0)	375 (54.4)	974 (56.7)
Severe	546 (39.9)	563 (40.9)	293 (42.5)	701 (40.8)
Very severe	22 (1.6)	21 (1.5)	13 (1.9)	28 (1.6)
Severity at baseline, n (%)				
Mild	0 (0.0)	1 (0.04)	0 (0.0)	1 (0.01)
Moderate	1879 (68.7)	1846 (67.2)	920 (66.9)	4645 (67.7)
Severe	858 (31.3)	901 (32.8)	456 (33.1)	2215 (32.3)

Mean age at onset based on data from ^a^1358 patients, ^b^1364 patients, ^c^684 patients, ^d^1703 patients.

AAC = acetylsalicylic acid, acetaminophen, caffeine; APAP = acetaminophen; SD = standard deviation.

### Pain-free after 2 h (primary endpoint)

The proportion of all treated headache episodes that were pain-free 2 h following treatment with AAC (28.5%) was significantly higher compared with APAP (21.0%) and placebo (18.0%) (p < 0.0001 vs. either) (see Additional file 1, Figure 3). APAP was also significantly superior to placebo (p = 0.007).

**Figure 3:**
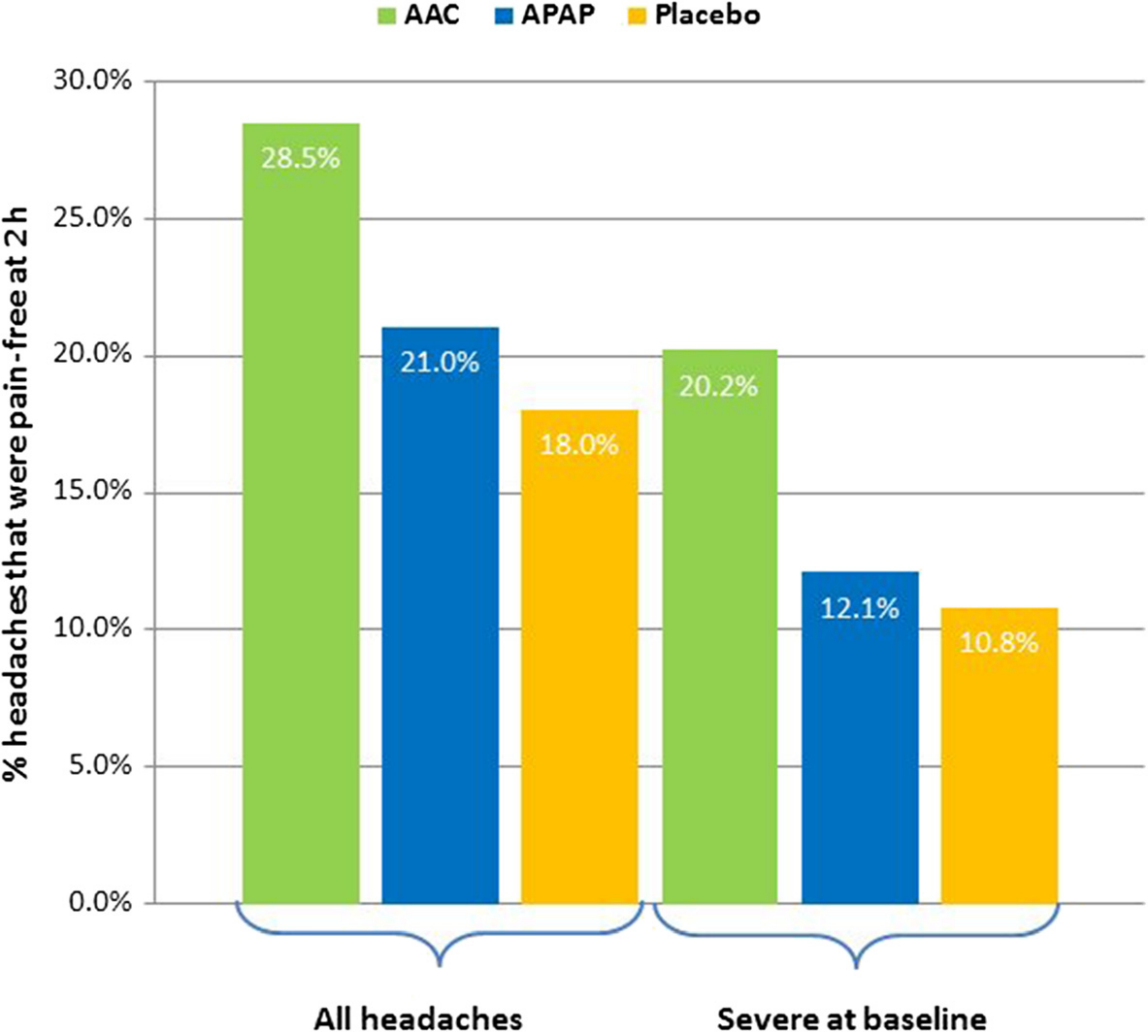
**Proportion of all headache episodes and severe headache episodes at baseline that were pain-free at 2 h after treatment.** AAC was significantly superior to APAP and placebo for all headache episodes (p < 0.0001 vs. both) and those that were severe at baseline (p < 0.0001 vs. APAP and p = 0.0003 vs. placebo). APAP was significantly superior to placebo for all headache episodes (p = 0.007) but not those that were severe at baseline (p = 0.71).

In the subgroup of treated headache episodes rated severe at baseline, a similar outcome was observed (Figure 3). The proportion of headache episodes that were pain-free 2 h following treatment with AAC (20.2%) was significantly superior to APAP (12.1%; p < 0.0001) and placebo (10.8%; p = 0.0003). However, APAP did not differ significantly from placebo (p = 0.71).

### Pain-free after 1 h

The proportion of all treated headache episodes that were pain-free 1 h following treatment with AAC (8.6%) was significantly higher compared with APAP (6.1%; p = 0.0004) and placebo (5.4%; p = 0.019) (see Additional file 1). There was no significant difference between APAP and placebo (p = 0.98).

In the subgroup of treated headache episodes with pain rated severe at baseline, a similar outcome was observed. The proportion of headache episodes that were pain-free 1 h following treatment with AAC (6.5%) was significantly superior to APAP (3.9%; p = 0.0008) and placebo (3.1%; p = 0.015). However, APAP did not differ significantly from placebo (p = 0.95).

### Headache response after 2 h

The proportion of all treated headache episodes that had responded 2 h following treatment with AAC (66.6%) was significantly higher compared with APAP (58.2%) and placebo (48.8%) (p < 0.0001 vs. either) (see Additional file 2, Figure 4). APAP was also significantly superior to placebo (p < 0.0001).

**Figure 4:**
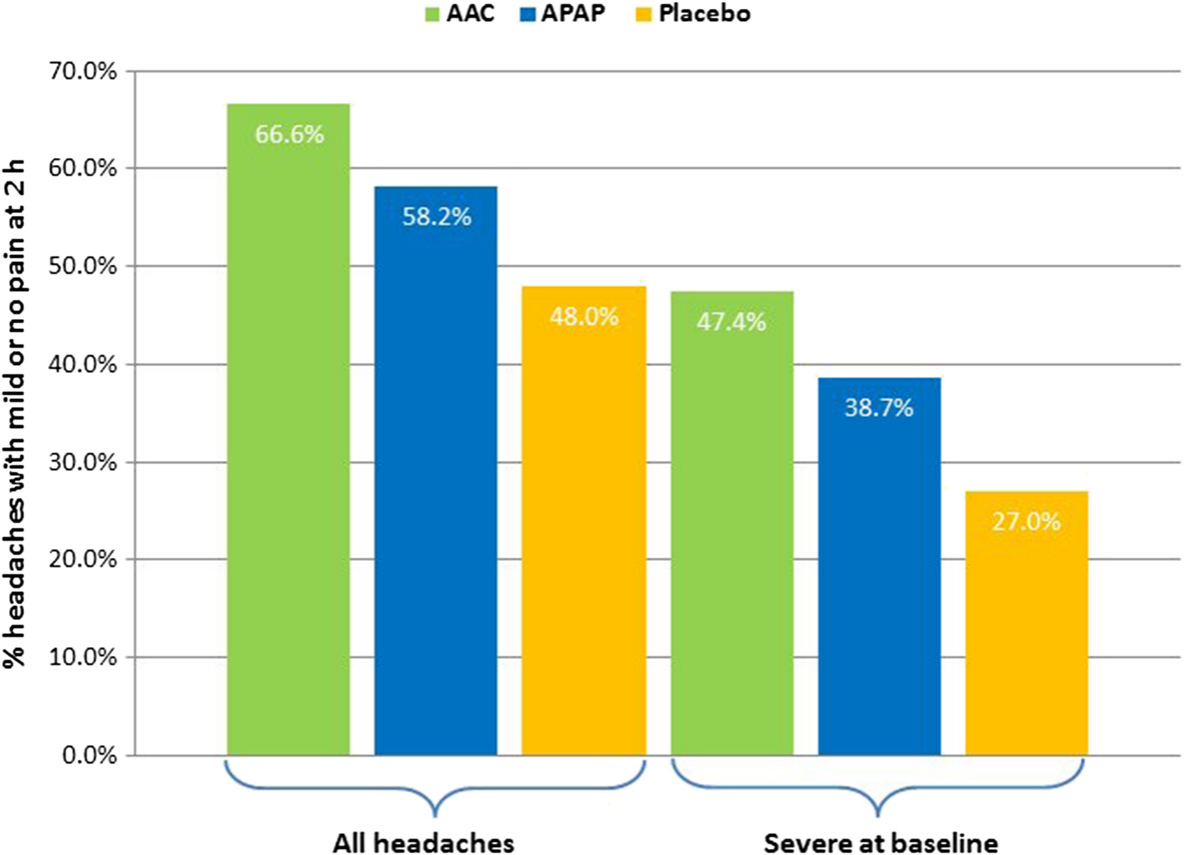
**Proportion of all headache episodes and severe headache episodes at baseline with mild or no pain at 2 h after treatment.** AAC was significantly superior to APAP and placebo for all headache episodes (p < 0.0001 for both) and for those that were severe at baseline (p = 0.0002 vs. APAP and p < 0.0001 vs. placebo). APAP was significantly superior to placebo (p < 0.0001) for all headache episodes and those that were severe at baseline (p = 0.003).

In the subgroup of treated headache episodes with pain rated severe at baseline, a similar outcome was observed. The proportion of headache episodes that had responded 2 h following treatment with AAC (47.4%) was significantly superior to APAP (38.7%; p = 0.0002) and placebo (27.0%; p < 0.0001) (Figure 4). APAP was also significantly superior to placebo (p = 0.003).

### Degree of interference with daily activities

Over the set of all treated headache episodes in the EE population, daily activities were rated ‘no more difficult than normal’ as early as 1 h after dosing in 30.2% of headaches treated with AAC, increasing to 76.1% by 4 h (Table 2). These results were significantly superior to the corresponding results with APAP (25.8% at 1 h, increasing to 69.6% at 4 h; p ≤ 0.0005) and placebo (22.8% at 1 h, increasing to 60.9% at 4 h; p ≤ 0.0001) (Table 2). APAP was significantly superior to placebo from 2–4 h (p ≤ 0.02).

**Table 2:** Proportion of patients who found daily activities to be ‘no more difficult than normal’ after treatment

**Treatment**	**Proportion (%) of patients who found daily activities to be ‘no more difficult than normal’ after treatment**											
	**1 h**	**p-value**		**2 h**	**p-value**		**3 h**	**p-value**		**4 h**	**p-value**	
All headache episodes												
AAC (n = 2734 – 2736 over 1 h – 4 h)	30.2	AAC vs. P	0.0001	47.8	AAC vs. P	<0.0001	64.8	AAC vs. P	<0.0001	76.1	AAC vs. P	<0.0001
APAP (n = 2738 – 2747 over 1 h – 4 h)	25.8	A vs. P	0.16	41.3	A vs. P	0.016	57.7	A vs. P	<0.0001	69.6	A vs. P	<0.0001
Placebo (n = 1367 – 1375 over 1 h – 4 h)	22.8	AAC vs. A	0.0005	36.3	AAC vs. A	<0.0001	51.0	AAC vs. A	<0.0001	60.9	AAC vs. A	<0.0001
Severe at baseline												
AAC (n = 856 – 858 over 1 h – 4 h)	17.1	AAC vs. P	0.18	31.1	AAC vs. P	0.079	48.1	AAC vs. P	0.0001	62.5	AAC vs. P	<0.0001
APAP (n = 897 – 901 over 1 h – 4 h)	13.4	A vs. P	0.93	27.2	A vs. P	0.60	43.1	A vs. P	0.046	56.5	A vs. P	0.032
Placebo (n = 453 – 456 over 1 h – 4 h)	10.5	AAC vs. A	0.033	20.4	AAC vs. A	0.082	34.0	AAC vs. A	0.011	45.7	AAC vs. A	0.002

AAC = acetylsalicylic acid, acetaminophen, caffeine; A or APAP = acetaminophen; P = placebo.

In the subgroup of treated headache episodes with pain rated severe at baseline, the proportion in which daily activities were rated as ‘no more difficult than normal’ was significantly higher after treatment with AAC vs. APAP at 1, 3 and 4 h (difference of 3.7 percentage points at 1 h and 6 percentage points at 4 h, p ≤ 0.033) and vs. placebo at 3 and 4 h (difference of 14.1 percentage points at 3 h and 16.8 percentage points at 4 h, p ≤ 0.0001) (Table 2). APAP was also significantly superior to placebo at 3 and 4 h (difference of 9.1 percentage points at 3 h and 10.8 percentage points at 4 h, p ≤ 0.046).

### Safety profile

According to the analysis by Migliardi et al. [10], there was a greater incidence of any AE (17%) compared with APAP alone (10%) or placebo (9%) (Table 3). Most AEs could be grouped into the categories of ‘stomach discomfort’, ‘nervousness’ and ‘dizziness’; other AEs were approximately equally distributed across the three treatment groups. None of the AEs were serious, and all were transitory.

**Table 3:** **Incidence of adverse events in the safety population (i.e. all subjects who dosed at least once with any study medication) in the four randomized, controlled studies (reproduced with permission from Migliardi et al.)**[10]

**Adverse event**	**Number of patients, n (%)**		
	**AAC (n = 1400)**	**APAP (n = 1400)**	**Placebo (n = 702)**
Any adverse event	241 (17)	136 (10)	61 (9)
Stomach upset	130 (9)	67 (5)	34 (5)
Nervousness	61 (4)	13 (1)	4 (1)
Dizziness	58 (4)	22 (2)	7 (1)

AAC = acetylsalicylic acid, acetaminophen, caffeine; APAP = acetaminophen.

## Discussion

This post-hoc meta-analysis employed new efficacy endpoints to update the results of four randomized studies that were performed in 1986. These endpoints (i.e. pain-free and headache response after 2 h) have been recommended to better differentiate among treatments in clinical trials of TTH [11,12]. Results demonstrate that AAC is effective in the treatment of episodic TTH, and significantly superior to APAP with respect to being pain-free at 2 h, having headache response at 2 h and being able to return to daily activities. The beneficial effects on pain are in agreement with other studies; the reduction in headache pain was greater with the triple combination vs. monotherapy, using lower doses of analgesic [9,15]. However, the double combination of APAP plus caffeine was not effective than monotherapy with naproxen [16].

Migliardi et al. [10] found that the triple combination is well tolerated, with AEs that were consistent with the known profile of the drug [10]. It was speculated that the greater incidence of ‘nervousness’ and ‘dizziness’ could be attributed to the caffeine content; on the other hand, the greater incidence of ‘stomach discomfort’ with AAC was probably caused by the presence of ASA. In contrast, the study by Diener at al. [9] included a subgroup of patients with episodic TTH; although the results of this subgroup were not reported, the triple combination was well tolerated in episodic TTH, and did not result in more AEs than monotherapy (HC Diener, unpublished data, [9]). A favorable risk-benefit ratio was also observed in a previous study that used this triple combination in TTH, albeit with a lower dose of caffeine (50 mg) [15].

Clearly, the combination offers an important alternative when APAP alone is not effective enough. Caffeine, in particular, contributes to the greater efficacy of the combination vs. APAP alone; patients with TTH or other pain conditions who take an analgesic without caffeine need about 40% more medication to get the same relief as patients taking the same analgesic with caffeine [17]. Analgesic combinations containing caffeine have been recommended as first-line [18] or Level I [19] therapies to manage episodic TTH.

In our pooled population, over 30% of treated headaches had pain rated severe at baseline – much higher than reported by Rasmussen et al. [20]. Nevertheless, several other studies in episodic TTH have reported comparably higher proportions of episodic TTH patients with severe pain [16,21,22]. However, we cannot absolutely exclude the possibility that some of the treated headaches were migraine attacks, as it is not clear whether associated symptoms were recorded. The phenotype of treated headache attacks might have deviated from the diagnosis made by prior history. This problem, inherent to studies of this type, has been noted previously [23]. Nevertheless, subjects were only included in the studies if they met criteria that adhered to the definition of TTH at the time, matching at least two of the criteria of episodic TTH used today [14]. In addition, patients were excluded if they had a history of “vascular headache of migraine type” – although it should be noted that prior to inclusion in the studies, headache attacks were described as throbbing (or a combination of throbbing/non-throbbing) in some patients. This type of ‘pulsating’ pain is more commonly associated with migraine, however the IHS classification does not preclude it as a characteristic of episodic TTH [14]. Therefore, it is not unreasonable to assume that the majority of ‘severe’ headaches were in fact tension-type.

There is also a common perception that the pain associated with episodic TTH can only be mild to moderate. However, while it is true that the IHS states that the pain intensity associated with episodic TTH is *typically* mild to moderate (particularly in contrast to the pain of a migraine, for example, which is classed as moderate to severe), the criteria do not specify that the pain *has* to be mild to moderate [14]. Only two of the four characteristics need to be met to diagnose episodic TTH (i.e. bilateral location, pressing/tightening (non-pulsating) quality, mild or moderate intensity, not aggravated by routine physical activity such as walking or climbing stairs [14]), so the pain might indeed be severe. Especially considering that the way in which patients rate the severity of their pain can vary considerably, depending on perception – each patient has different pain thresholds and moderate pain in one patient may be regarded as severe by another. In the studies in our meta-analysis, the patients rated their own pain and may simply have perceived it to be severe, whereas a physician might have rated it as moderate. Despite these uncertainties, our meta-analysis demonstrates that the triple combination was superior to monotherapy in the subset of headache episodes with severe pain at baseline; the proportion of severe headache episodes treated that were pain-free after 2 h was 67% higher with AAC compared with APAP.

Our meta-analysis also evaluated the impact of the triple combination on daily activities, an aspect that is rarely studied in the literature. Even mild episodic TTH can have an impact on cognition and attention, which can impair performance and successful completion of general tasks [24] and lead to reduced productivity [3,25,26]. Therefore, any analgesic that is effective in this regard will be of interest to patients. Our results demonstrate that daily activities were ‘no more difficult than normal’ from as little as 1 h after treatment with the triple combination (Table 2).

The strength of the present study is the sample size and the large number of treated headache episodes. The crossover design increases the power of the trial [27]. A limitation of the study is the long time period that has passed since these studies were performed. Consequently, operational criteria for the conduct and analysis of studies for the treatment of TTH were not available. Although the design limitations cannot be addressed at this late date, we consider them to have modest impact. In addition, it cannot be ignored that some of the headache attacks during the studies may have been migraine rather than episodic TTH, as noted above. Either way, the combination of ASA, APAP and caffeine is superior to APAP monotherapy and placebo even in patients with severe headache and is well tolerated.

## Conclusions

Pooled data from 6,861 treated headache episodes confirms the efficacy of the fixed combination of acetylsalicylic acid, acetaminophen and placebo in the treatment of episodic TTH. AAC was significantly superior to 1000 mg APAP and to placebo with respect to the percent pain-free at 2 h, as well as the percent with headache response (mild or no pain) at this point. Similar superiority was observed in the subset of treated headaches with pain rated severe at baseline. In addition, significantly more AAC patients were able to return to their daily activities 1 h after dosing vs. either 1000 mg APAP or placebo. AAC was generally well tolerated, with a predictable safety profile.

## Abbreviations

AAC: Acetylsalicylic acid, acetaminophen and caffeine; AEs: Adverse events; APAP: Acetaminophen; ASA: Acetylsalicylic acid; EE: Efficacy evaluable; NSAIDs: Non-steroidal anti-inflammatory drugs; OTC: Over-the-counter; TTH: Tension-type headache.

## Competing interests

HCD received honoraria for participation in clinical trials, contribution to advisory boards or oral presentations from: Addex Pharma, Allergan, Almirall, Autonomic Technology, AstraZeneca, Bayer Vital, Berlin Chemie, Böhringer Ingelheim, Bristol-Myers Squibb, Coherex, CoLucid, Electrocore, GlaxoSmithKline, Grünenthal, Janssen-Cilag, Lilly, La Roche, 3 M Medica, Medtronic, Menerini, Minster, MSD, Neuroscore, Novartis, Johnson & Johnson, Pierre Fabre, Pfizer, Schaper and Brümmer, Sanofi, St. Jude and Weber & Weber. Financial support for research projects was provided by Allergan, Almirall, AstraZeneca, Bayer, GSK, Janssen-Cilag, MSD and Pfizer. Headache research at the Department of Neurology in Essen is supported by the German Research Council (DFG), the German Ministry of Education and Research (BMBF) and the European Union. HCD has no ownership interest and does not own stocks of any pharmaceutical company.

MG is an employee of Novartis Consumer Health Inc., Parsippany, NJ, USA.

MH is an employee of Novartis Consumer Health S.A., Nyon, Switzerland.

## Authors’ contributions

H-CD participated in the design of the study and helped to draft the manuscript. MG participated in the design of the study, performed all analyses and helped to draft the manuscript. MH conceived of the study, participated in its design and coordination, and helped to draft the manuscript. All authors read and approved the final manuscript.

## Supplementary Material

Additional file 1 Proportion of headache episodes that were pain-free at each hourly assessment after treatment. Click here for file

Additional file 2 Proportion of headache episodes with mild or no pain at each hourly assessment after treatment. Click here for file
