# Supplementary material for: Use of a fixed combination of acetylsalicylic acid, acetaminophen and caffeine compared with acetaminophen alone in episodic tension-type headache: meta-analysis of four randomized, double-blind, placebo-controlled, crossover studies
Source: J Headache Pain. 2014 Nov 19;15(1):76. doi: 10.1186/1129-2377-15-76 (PMC4256978; doi:10.1186/1129-2377-15-76)
Supplement: Additional file 1 — Proportion of headache episodes that were pain-free at each hourly assessment after treatment. [file 1129-2377-15-76-S1.docx]

**Proportion of headache episodes that were pain-free at each hourly assessment after treatment**

| Treatment | Proportion (%) of headache episodes treated that were pain-free after treatment | | | | | | | | | | | |
| --- | --- | --- | --- | --- | --- | --- | --- | --- | --- | --- | --- | --- |
|  | 1 h | p-value | | 2 h | p-value | | 3 h | p-value | | 4 h | p-value | |
| All headache episodes | | | | | | | | | | | | |
| AAC (n=2737) | 8.6 | AAC vs. P | 0.019 | 28.5 | AAC vs. P | <0.0001 | 49.3 | AAC vs. P | <0.0001 | 65.9 | AAC vs. P | <0.0001 |
| APAP (n=2748) | 6.1 | A vs. P | 0.98 | 21.0 | A vs. P | 0.007 | 40.1 | A vs. P | <0.0001 | 57.0 | A vs. P | <0.0001 |
| Placebo (n=1376) | 5.4 | AAC vs. A | 0.0004 | 18.0 | AAC vs. A | <0.0001 | 32.1 | AAC vs. A | <0.0001 | 48.3 | AAC vs. A | <0.0001 |
| Severe at baseline | | | | | | | | | | | | |
| AAC (n=858) | 6.5 | AAC vs. P | 0.015 | 20.2 | AAC vs. P | 0.0003 | 37.4 | AAC vs. P | <0.0001 | 52.5 | AAC vs. P | <0.0001 |
| APAP (n=901) | 3.9 | A vs. P | 0.95 | 12.1 | A vs. P | 0.71 | 27.4 | A vs. P | 0.017 | 44.5 | A vs. P | 0.0008 |
| Placebo (n=456) | 3.1 | AAC vs. A | 0.0008 | 10.8 | AAC vs. A | <0.0001 | 20.4 | AAC vs. A | 0.0001 | 35.3 | AAC vs. A | 0.0002 |

*AAC=acetylsalicylic acid, acetaminophen, caffeine; A or APAP=acetaminophen; P=placebo*
