# Supplementary material for: Use of a fixed combination of acetylsalicylic acid, acetaminophen and caffeine compared with acetaminophen alone in episodic tension-type headache: meta-analysis of four randomized, double-blind, placebo-controlled, crossover studies
Source: J Headache Pain. 2014 Nov 19;15(1):76. doi: 10.1186/1129-2377-15-76 (PMC4256978; doi:10.1186/1129-2377-15-76)
Supplement: Additional file 2 — Proportion of headache episodes with mild or no pain at each hourly assessment after treatment. [file 1129-2377-15-76-S2.docx]

**Proportion of headache episodes with mild or no pain at each hourly assessment after treatment**

| Treatment | Proportion (%) of headache episodes that responded to treatment | | | | | | | | | | | |
| --- | --- | --- | --- | --- | --- | --- | --- | --- | --- | --- | --- | --- |
|  | 1 h | p-value | | 2 h | p-value | | 3 h | p-value | | 4 h | p-value | |
| All headache episodes | | | | | | | | | | | | |
| AAC (n=2737) | 40.0 | AAC vs. P | <0.0001 | 66.6 | AAC vs. P | <0.0001 | 82.9 | AAC vs. P | <0.0001 | 89.1 | AAC vs. P | <0.0001 |
| APAP (n=2748) | 33.7 | A vs. P | <0.0001 | 58.2 | A vs. P | <0.0001 | 75.3 | A vs. P | <0.0001 | 83.2 | A vs. P | <0.0001 |
| Placebo (n=1376) | 27.4 | AAC vs. A | <0.0001 | 48.8 | AAC vs. A | <0.0001 | 66.8 | AAC vs. A | <0.0001 | 74.2 | AAC vs. A | <0.0001 |
| Severe at baseline | | | | | | | | | | | | |
| AAC (n=858) | 23.0 | AAC vs. P | <0.0001 | 47.4 | AAC vs. P | <0.0001 | 68.5 | AAC vs. P | <0.0001 | 81.5 | AAC vs. P | <0.0001 |
| APAP (n=901) | 16.2 | A vs. P | 0.16 | 38.7 | A vs. P | 0.003 | 60.2 | A vs. P | 0.0009 | 74.4 | A vs. P | 0.0007 |
| Placebo (n=456) | 10.8 | AAC vs. A | 0.0001 | 27.0 | AAC vs. A | 0.0002 | 46.5 | AAC vs. A | 0.001 | 60.1 | AAC vs. A | 0.002 |

*AAC=acetylsalicylic acid, acetaminophen, caffeine; A or APAP=acetaminophen; P=placebo*
